# Improved Manufacturing Performance of Screen Printed Carbon Electrodes through Material Formulation

**DOI:** 10.3390/bios6030030

**Published:** 2016-06-27

**Authors:** Eifion Jewell, Bruce Philip, Peter Greenwood

**Affiliations:** SPECIFIC, College of Engineering, Swansea University, Fabian Way, SA1 8AN Swansea, UK; b.philip@swansea.ac.uk (B.P.); p.greenwood@swansea.ac.uk (P.G.)

**Keywords:** screen printing, carbon electrodes, manufacturing

## Abstract

Printed carbon graphite materials are the primary common component in the majority of screen printed sensors. Screen printing allows a scalable manufacturing solution, accelerating the means by which novel sensing materials can make the transition from laboratory material to commercial product. A common bottleneck in any thick film printing process is the controlled drying of the carbon paste material. A study has been undertaken which examines the interaction between material solvent, printed film conductivity and process consistency. The study illustrates that it is possible to reduce the solvent boiling point to significantly increase process productivity while maintaining process consistency. The lower boiling point solvent also has a beneficial effect on the conductivity of the film, reducing the sheet resistance. It is proposed that this is a result of greater film stressing increasing charge percolation through greater inter particle contact. Simulations of material performance and drying illustrate that a multi layered printing provides a more time efficient manufacturing method. The findings have implications for the volume manufacturing of the carbon sensor electrodes but also have implications for other applications where conductive carbon is used, such as electrical circuits and photovoltaic devices.

## 1. Introduction

Screen printed carbon graphite pastes are the main materials used for bio sensors electrodes, [1,2,3,4]. From a research perspective carbon/graphite materials are considered commercially mature. In recent times little scientific research has been conducted on the impact of formulation on sensor performance, although comparisons between commercial materials have been carried out [5,6] which highlighted each material behaved differently. More recently, research focus has concentrated on more research attractive materials such as Carbon nano tubes CNTs [7], and multi walled carbon nano tubes MWCNTs, Graphene and their hybridization with conventional graphite materials, [8]. The limited formulation studies have focused on the influence of the carbon/graphite relative proportions, the carbon/graphite physical and electrical characteristics and the proportion of conductive material required for charge percolation through the cured film [9]. A variety of binder systems are compatible with carbon/graphite materials, such as, epoxies, alkyds, acrylics, vinyls, polyurethane resins or phenolic resins and the choice is governed by the curing temperature, substrate which is being printed, film flexibility and the resin compatibility with the final application. The choice of binder/binders is usually proprietary information held by manufacturers.

The beauty of the screen printing process is that it is equally suited to small batch laboratory testing as to volume manufacturing, making the process of upscaling manufacture less problematic. However, there remains significant challenges in transitioning a laboratory to manufacturing process and the solvent choice plays an important role in this transition. The role of the solvent is to dissolve the binder, control the formulation viscosity and control the drying and curing process. A common bottleneck in many printing processes is the drying and curing process, which has a subsequent effect on productivity and ultimately profitability. Given the thick film nature of the screen printing process, a more volatile solvent would seem preferable as it potentially improves process productivity. A more volatile solvent may however also result in reduced process consistency as it evaporates during the printing processing. This can lead to “drying in”, the process where the ink begins to dry in the mesh, which is a particular issue for finer detail printing. A more volatile solvent also leads to increased viscosity variation and as the process is viscosity dependent [10], this can lead to variation in print quality. A further solvent effect on process is that the migration of the solvent into the polyurethane squeegee can impact the squeegee properties and hence impact process consistency [11,12]. During material formulation, a compromise must be made between solvent volatility, which is essentially a balance between productivity (which demands low boiling points) versus consistency (which requires a high boiling point). There is therefore an interaction between solvent properties, printed electrode characteristics and process productivity at volume. The solvent volatility has been shown to play role in film stresses induced during drying, [13], with faster evaporation leading to greater stress in the film [14]. The impact of this residual stress on conductivity of carbon materials has not been reported publically. In carbon graphite, there is a dearth of information on the effect of solvent on the performance of the cured film and the ease of processing. Given that the volume production is a primary advantage of screen printing, then this is worthy of investigation.

The aim of the study was to understand the impact of solvent on the screen printed curing performance of the carbon graphite conductive nitrocellulose (NC) based materials and to establish the role of the solvent on performance, process consistency and stability. This information would allow material formulators to estimate the balance of solvents and allow sensor manufacturers to estimate the impact of formulation changes on their process.

## 2. Materials and Methods

The materials were manufactured by Gwent Electronic Materials using the same carbon graphite/carbon black proportion, the same nitrocellulose binder and with the same solvent mass proportion. The net result was four materials whose constituents were identical in constituent parts apart from the solvent. The solvents and solvent blends were chosen from commercially viable solvents (health and safety compliant and cost) and on the basis of their relative boiling point. The boiling point being the characteristic that largely dictates the drying and in process variation. The final composition of the material is shown in Table 1.

Each material was printed through five screens (Table 2) in order to examine the interaction of the film thickness and solvent on the curing characteristics. The screens represent a range which is applicable to the production of carbon electrode sensors. The theoretical volume represents the geometric free air volume available within the mesh and is an indicator of film thickness only. The material was printed to a polyester coated steel substrate which was chosen as it provided a surface which was compatible with the material and general sensor manufacturing but also facilitated a structurally stable base substrate which would not undergo any form of deformation at high temperatures. Printing was carried out on an ATMA AT-25PA screen printer with drying taking place in a 3 m Thieme conveyor tunnel dryer operating at 1.2 m/min, a residence time of 2.5 min. An air temperature of 155 °C was used to provide a favourable environment for substrate heating and mass transfer from the coating, with air impinged at 155 °C to the surface from an array of holes. Multiple passes were made through the tunnel dryer with electrical performance being monitored at each intermediate stage on a cooled substrate.

Electrical performance was characterised by sheet resistance which was measured using 4-point sheet resistance with 2 mm electrode spacing and the subsequent sheet resistance was calculated using constants which reflect the geometric sizes being measured, [15]. Three measurements were made on each of five substrates. Within the sample size of 15 measurement points, the uncertainty in the sheet resistance was calculated to be ± 1 Ω/sq. In addition to the sheet resistance, two point resistance measurements were carried out on a spiral structure containing 56 cm of line of 700 μm width. In this way, the impact on fine structures, which are more relevant to sensor electrode design and manufacture, could also be analysed. Film thickness measurements were carried out using an Elcometer 456 induction gauge which was zeroed on the base polyester coating.

Thermogravimetric analysis (TGA) was used to establish the relative evaporative characteristics of the materials once formulated, which when combined with rheological data would provide an indicator of manufacturing capability. TGA analysis was carried out using a Perkin Elmer Pyris 1 analysis in a nitrogen atmosphere. TGA isotherms were carried out at 130 °C and 155 °C as these represented the mean substrate temperature experienced during the substrate passage through the dryer (as measured by contact thermocouples) and the set air impingement temperature respectively. The true material exposure temperature therefore lies between these limits. Material rheology was carried out using a Brookfield RS cone and plate viscometer at 20 °C operating through a constant stress shear ramp from 5 to 500 Pa with 50 intermediate steps.

## 3. Results

### 3.1. Material Characteristics

**Figure 1:**
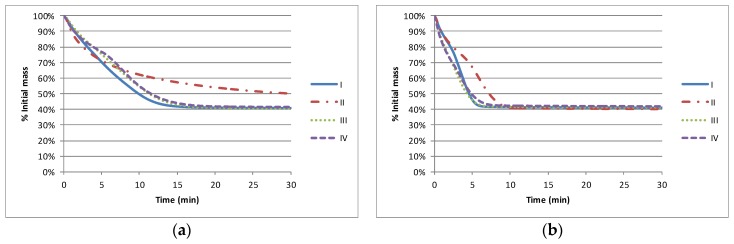
Thermogravimetric analysis (TGA) analysis of the materials at an isothermal temperature of (**a**) Mean substrate temperature of 130 °C and (**b**) Impingement air temperature of 155 °C.

The impact of the solvent on the nominal drying characteristics (as measured by TGA) is shown in Figure 1. Where there is a constituent of lower boiling point material, (I, II and IV), all solvent is lost (40% solids) by approximately 15 min at 130 °C and by 6 min at 155 °C. The higher boiling point material (II) does not achieve its stable solids level of 40% within 30 min at 120 °C and takes approximately 10 min to achieve the solids level at 155 °C.

**Figure 2:**
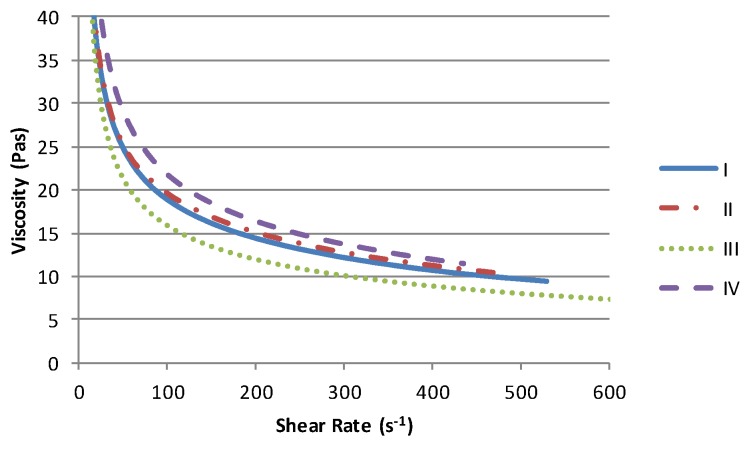
Controlled stress viscosity profiles for each material (50 points on each curve).

The solvent has a minimal effect on the form of the conductive paste viscosity profile, Figure 2. Each exhibits pseudoplastic behaviour with low shear viscosities upwards of 40 PaS. Given that there is an order of magnitude difference in the viscosity of the solvent (Table 2) and that this is the primary constituent of each material (60%), then the viscosity range of 5 PaS can be considered relatively small in comparison to the reduction in viscosity with increasing shear. Within this range the materials containing the high viscosity Terpineol solvent (II & IV) produce the highest viscosities while the lowest viscosity is obtained with lowest viscosity solvent (III).

**Figure 3:**
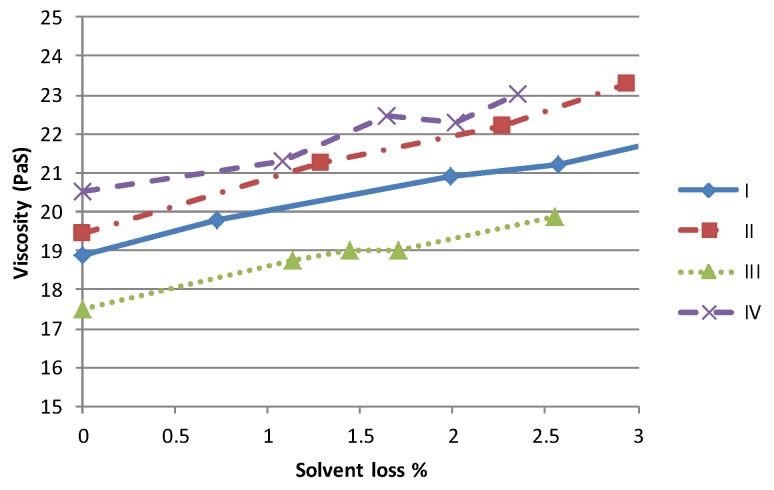
Simulation of the effect of solvent loss on material viscosity at a shear rate of 100 s^−1^.

One of the primary impacts of a higher volatility solvent is to accelerate the rate of viscosity variation due to solvent evaporation. In order to simulate the effect of changes in material rheology during the printing process, known quantities of each material were left to evaporate solvent and variations in mass and viscosity due to solvent loss were measured at regular intervals. As the form of each viscosity/shear rate curve is similar the impact of solvent loss is best examined by studying the viscosity at a given shear rate. At 100 s^−1^, each material shows a near linear relationship between the solvent lost and the viscosity, Figure 3. The limit of 3% mass loss was imposed as the samples of higher viscosity material were felt to have increased beyond that which could be printed successfully. Although the behaviour of each material is similar, clearly the time required to achieve the same mass loss would be lower for the more volatile materials, for a given set of operating conditions.

### 3.2. Printed Characteristics

**Figure 4:**
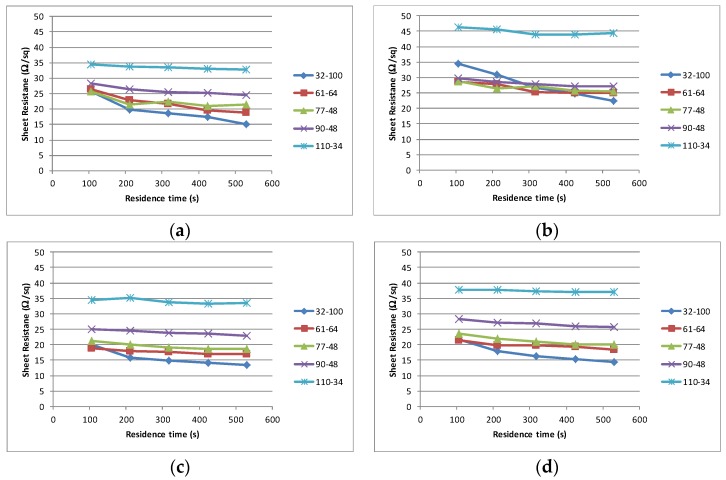
Sheet resistance/residence time profiles for each solvent (I to IV) printed through each screen. (**a**) I; (**b**) II; (**c**) III; (**d**) IV.

Having established the properties of the materials, each sample was subjected to an identical printing process. All materials printed well, exhibiting excellent adhesion and dried without any defects such film cracking, delamination or mottling. The interaction between the mesh type used and the solvent volatility on the curing time and electrical performance of the cured film is shown in Figure 4. In all instances the finest mesh 110–34 produces the highest sheet resistance, although in each instance the change in sheet resistance is minimal with increased residence time (successive passes through the dryer). As the mesh coarseness increases the sheet resistance drops and the time taken to achieve the stable sheet resistance increases. In all cases, the coarsest mesh (32–100) does not achieve a stable value with a residence time of 530 s.

**Figure 5:**
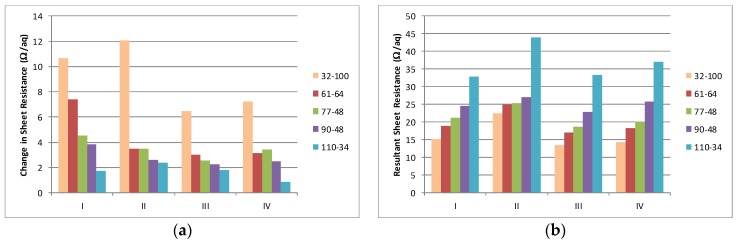
(**a**) Change in sheet resistance and (**b**) Minimum sheet resistance for a residence time of 530 s for each material through each mesh.

The interaction between the solvent type and the change in resistance over the maximum residence time of 530 s is summarised in Figure 5a. The final resistance achieved after the maximum residence time illustrates that the coarsest (32–100) produces the lowest sheet resistance and that, in most instances, the sheet resistance increases in a step wise manner as the mesh becomes finer, Figure 5b. The change in sheet resistance is most evident with the coarsest mesh (32–100).

**Figure 6:**
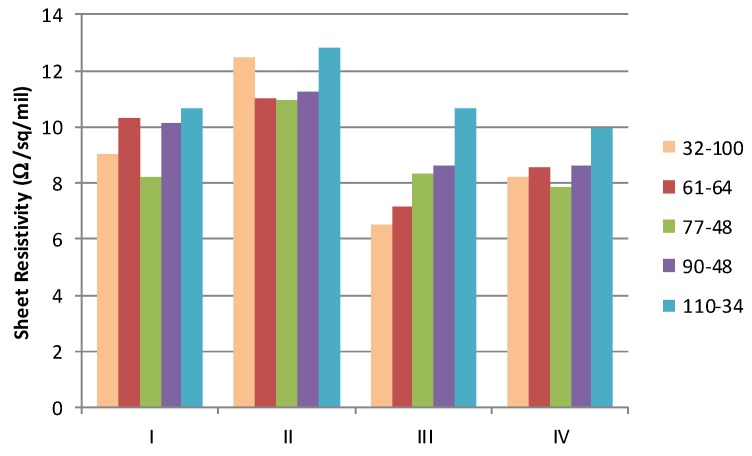
Normalised sheet resistivity (expressed in Ω/sq/mil) for each mesh ink formulation.

When the conductivity of the cured materials is normalised using standard industry notation (Ω/sq/mil where 1 mil = 25.4 μm), it is possible to examine the impact of the mesh on the sheet resistance, Figure 6. If the deposit of the material was consistent and the film form and conductive element distribution were equal, then this normalised sheet resistivity should be independent of the mesh used. However, materials with the more volatile solvents (I, III and IV) exhibit lower sheet resistivity for the coarsest screens (thicker films) than with those with finer screens. It is postulated that this is associated with some filtering by the finer meshes which reduce the larger particle graphite component in the film.

When the resistivity is subsequently compared to that of similar commercial materials, it is shown that the formulation presents an improvement over that which is available commercially. A review of commercial materials indicates that the minimum available sheet resistivity is around 14 Ω/sq/mil. The material described therefore represents approximately a 50% reduction in material resistivity.

**Figure 7:**
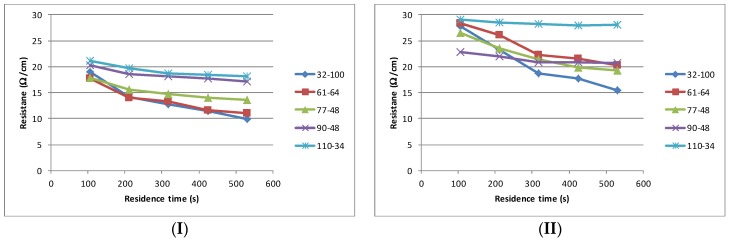

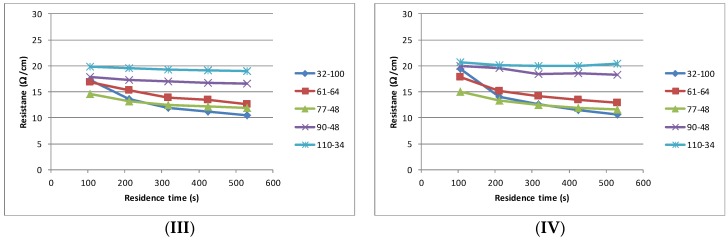
Resistance (expressed in Ω/ linear cm) for a 700 μm line—curing time profile for each of the material (**I** to **IV**) and mesh combinations.

While sheet resistance is an excellent measure of material, mesh and curing performance most electrodes are made from discrete geometric structures and thus a study of carbon electrode performance also needs to examine the printing of such structures. The resistance/curing characteristics of the structures closely follows that which is seen with the sheet resistance, Figure 7. Additional drying time has a negligible effect on the resistance for the finest mesh (110–34) but has an increasing effect as the mesh coarseness (film thickness) is increased. The materials containing low boiling point solvents (I, III and IV) produced the lowest line resistance, with values typically between 10 Ω/cm and 20 Ω/cm for the coarsest and finest mesh. The higher boiling point material (II) consistently reports the highest line resistance.

### 3.3. Process Consistency Characteristics

**Figure 8:**
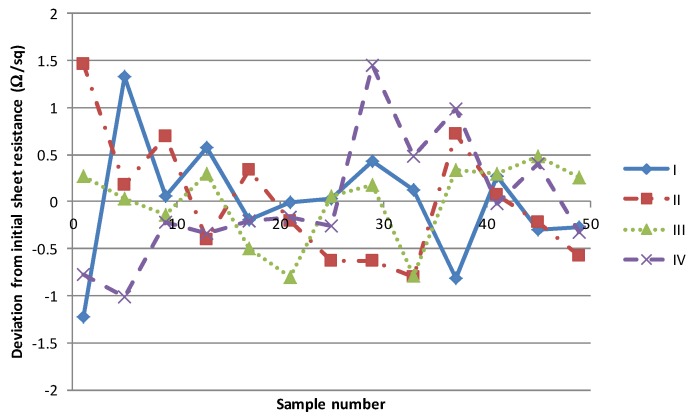
Variation observed in a short pilot run of 50 sheets.

As highlighted in the introduction, the primary effects of the solvent on the process consistency are associated with solvent evaporation altering material rheology and solvent absorption into the squeegee. In order to investigate the former, a short production of 50 samples of each material was carried out at a production rate of 30 s (total processing time of 25 min). These were given the maximum residence time of 530 s through the dryer in a single pass by retarding the speed of the dryer conveyor. The sheet resistance of every 5th sample was measured and the deviation from the mean starting value is illustrated in Figure 8. There is no clear trend in the measured sheet resistances with the variation observed deviating no more than ± 1.5 Ω/sq from a nominal mean. Given a measurement uncertainty of ± 1 Ω/sq then this can safely be assumed to be no more than natural process fluctuation.

**Figure 9:**
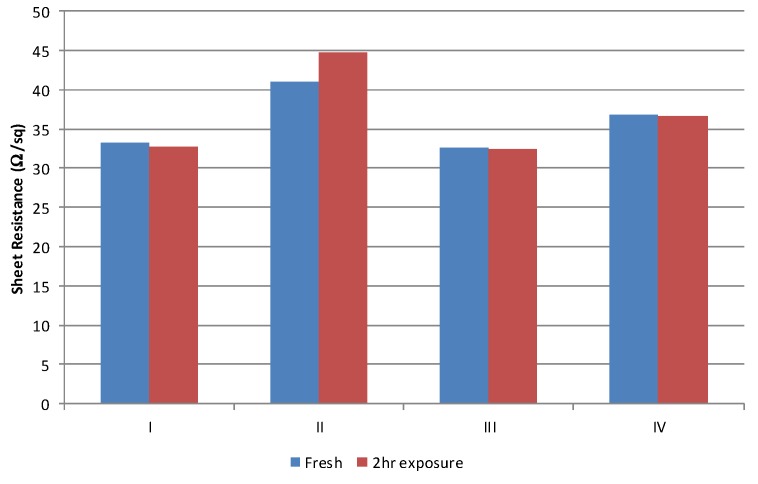
Sheet resistance of each material for a fresh and then a squeegee which has been exposed in the material for 2 h.

The other primary impact of the solvent on process consistency is associated with the absorption of the solvent into the polyurethane squeegee. This was established by cutting two fresh squeegee lengths, the edge of the first was placed in 10 mm of wet conductive material for 2 h. At the end of a 2 h period the squeegee which had been placed in the conductive carbon material was used to print five samples and then immediately replaced with the dry length, to print a further five samples. Thus, any changes in deposit can be attributed directly to the changes in squeegee properties due to absorption of materials in the ink. Exposure to the material resulted in a negligible difference over the 2 h period, Figure 9. There is an increase in sheet resistance of around 3 Ω/sq. for the material formulated with the type II solvent. This is beyond that which can be attributed error (± 1 Ω/sq) and shows that there is some interaction between the solvent and the polyurethane material. The exact chemical and physical mechanism of this small change has not been investigated as the performance of this material was the poorest of the materials investigated (Figure 4, Figure 5, Figure 6 and Figure 7) and as such its use would not be pursued.

## 4. Discussion

This experimental study has raised a number of issues which are worthy of discussion. The sheet resistance is significantly lower than that achieved with comparable commercial materials which have been found to be at best 14 Ω/sq/mil. The nitrocellulose binder was able to flex without impact on the substrate.

**Figure 10:**
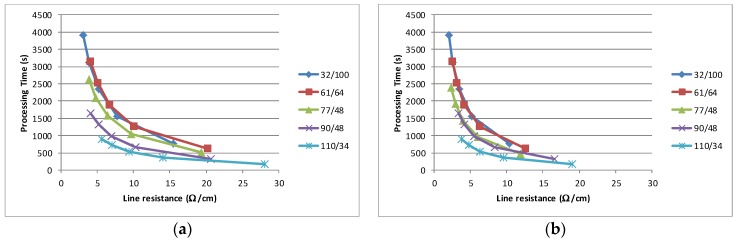
Simulated processing time/line resistance for a 700 μm wide line printed for solvent (II) and (III) for 1–5 layers of material. (**a**) Solvent II—Least conductive; (**b**) Solvent III—Most conductive.

The choice of solvent has been shown to have an impact on not only the drying but on the final conductivity of the film. Examined from a sheet resistance viewpoint, it has been possible to lower the thick film sheet resistance by 50% by changing from solvent II to solvent III (Figure 5b) and the thin film sheet resistance by 25% by changing from solvent II to solvent III (Figure 5b). It is proposed that the mechanism for this is associated with the higher volatility material producing increased tension within the film which leads to transverse compression in the cured film. This compression leads to greater inter particle contact, allowing easier charge transfer across the film. The result has implications for volume manufacturing, as for a given conductivity requirement (in terms of Ω/cm) the optimum processing characteristics can be calculated. Assuming a known production rate and a drying time required to reach within 5% of the final line resistance, then the relative merit of multiple thin layers as compared to a single thick layer can be established. This analysis was carried out for a printing time of 30 s per sheet (20 s for the loading in register and 5 s each for the printing and unloading) and a drying time to 95% conductivity, which are an integer multiple of a standard dryer pass of 150 s, i.e., if three passes were required to achieve conductivity which is 95% of the stabilised resistance, then the drying time is 450 s (3 × 150 s). The impact of the solvent is clearly shown in Figure 10 where this analysis has been carried out for the least and most conductive material. The net effect of the solvent is to lower the possible line resistance and to reduce the time required in order to achieve a given line resistance, e.g., a 5Ω/cm line could be a achieved by three layers through a 90/48 mesh in around 1000 s with the most conductive material, while the least conductive would take around 1400 s.

Each data point on every curve represents the number of layers which are printed, with a single layer at the highest resistance value and additional layers following the curve to lower line resistances. The analysis also illustrates that in all instances it is beneficial (from a processing time point of view) to deposit multiple layers instead of a single thick layer. As the thinner layers dry quickly, then the largest time element of the manufacturing process is reduced. Clearly there are simplifications in this analysis and the choice of processing time as the sole metric. The simplifications include; drying times can only be a multiple of a single pass, i.e., the drying speed cannot be varied, and the assumption that individual layers combine without any physical interaction. In terms of metric there is also a cost metric, e.g., there will be staff cost element for printing, while none is required for drying. In addition, engineering issues such as additional screen wear, increased “drying in” with finer meshes or difficulties in interlayer register may mean that the ideal manufacturing scenario differs from that predicted by the analysis. A more complex model should take these factors into account. Notwithstanding these issues, this processing analysis based on experimental results has demonstrated the principal that sequential multi-layer printing provides a more time efficient manufacturing method for carbon electrode sensors.

The primary metric used to characterise the materials during this study has been the sheet and line resistance. In order to fully link this to sensor performance, further studies should be carried out using the materials within a sensor structure. The work completed is equally applicable to other industries such as low temperature electrical circuits and the nascent flexible PV industry where carbon is being proposed as an electrode material, [16].

## 5. Conclusions

An experimental investigation on the effect of solvent on the performance of screen printed carbon materials and their process-ability has been undertaken. The study has identified that lower boiling point solvents can lead to more conductive films being produced, with significantly improved manufacturing throughput and no identifiable detrimental effect on process stability. It has also proposed a multi-layer approach to film deposition is a more time efficient means of producing high conductivity carbon paste films.

## Figures and Tables

**Table 1:** Material used in the experimental study.

	I	II	III	IV
C: Binder: Solvent wt %	31:9:60	31:9:60	31:9:60	31:9:60
Solvents	4-Hydroxy-4-Methyl-2 Pentanone (Diacetone alcohol)	P-Menth-1-En-8-ol (Terpineol)	2-Butoxyethanol	4-Hyrdroxy-4Methyl-2-Pentanone & P-Menth-1-En-8-ol (Terpineol)
Nominal solvent BP (°C)	166	219	171	166 & 219
Solvent viscosity (mPas) @20 °C	2.9	36	2.9	2.9 & 36

**Table 2:** Screens used in the experiment.

Notation	32–100	61–64	77–48	90–48	110–34
Mesh ruling (threads/cm)	32	61	77	90	110
Thread diameter (μm)	100	64	48	48	34
Mesh aperture size(μm)	209	90	77	55	54
Theoretical volume (cm^3^/m^2^)	73	30	27	19	19

## Abbreviations

The following abbreviations are used in this manuscript:

BP: Boiling point
